# Tetra­kis[1-phenyl-3-(1*H*-1,2,4-triazol-1-yl)propan-1-one-κ*N* ^4^]bis­(thio­cyanato-κ*N*)nickel(II)

**DOI:** 10.1107/S1600536810049081

**Published:** 2010-11-27

**Authors:** Jian-Hua Guo

**Affiliations:** a College of Chemistry, Tianjin Key Laboratory of Structure and Performance for Functional Molecules, Tianjin Normal University, Tianjin 300387, People’s Republic of China

## Abstract

In the centrosymmetric mononuclear title complex, [Ni(NCS)_2_(C_11_H_11_N_3_O)_4_], the Ni^II^ atom, located on an inversion centre, is hexa­coordinated in a distorted octa­hedral geometry comprising four N atoms of four monodentate 1-phenyl-3-(1*H*-1,2,4-triazol-1-yl)propan-1-one ligands and two N atoms from thio­cyanate anions.

## Related literature

Pseudohalide anions N_3_^−^, NCS^−^ and NCO^−^ are versatile ligands in coordination chemistry because of their multiple bridging modes, see: Yue *et al.* (2008 ▶). For a related structure, see: Guo & Cai (2007 ▶).
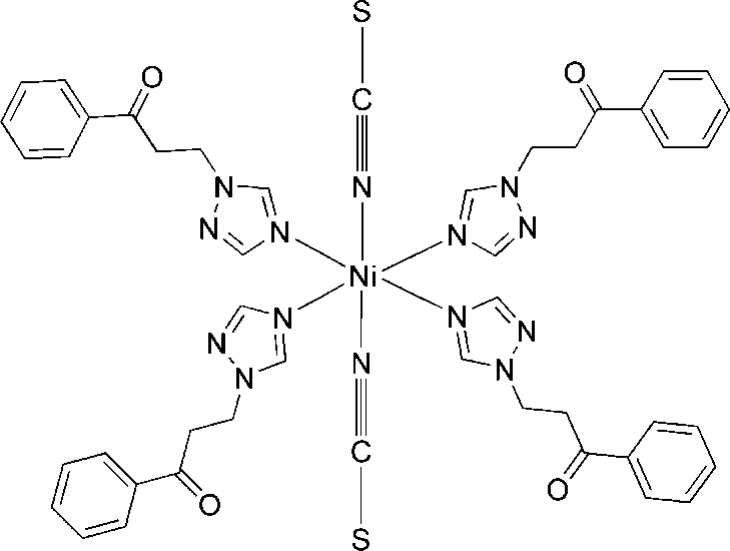


## Experimental

### 

#### Crystal data

[Ni(NCS)_2_(C_11_H_11_N_3_O)_4_]
*M* *_r_* = 979.78
Triclinic, 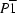

*a* = 7.8067 (10) Å
*b* = 11.8539 (15) Å
*c* = 13.8179 (17) Å
α = 68.907 (2)°
β = 74.765 (2)°
γ = 81.687 (2)°
*V* = 1149.3 (3) Å^3^
*Z* = 1
Mo *K*α radiation
μ = 0.57 mm^−1^
*T* = 293 K
0.32 × 0.28 × 0.22 mm

#### Data collection

Bruker APEXII CCD area-detector diffractometer
Absorption correction: multi-scan (*SADABS*; Sheldrick, 1996 ▶) *T*_min_ = 0.838, *T*_max_ = 0.884
6289 measured reflections
4016 independent reflections
3540 reflections with *I* > 2σ(*I*)
*R*_int_ = 0.015

#### Refinement

*R*[*F* ^2^ > 2σ(*F* ^2^)] = 0.029
*wR*(*F* ^2^) = 0.080
*S* = 1.06
4016 reflections
304 parameters
1 restraint
H-atom parameters constrained
Δρ_max_ = 0.38 e Å^−3^
Δρ_min_ = −0.34 e Å^−3^

### 

Data collection: *APEX2* (Bruker, 2003 ▶); cell refinement: *SAINT* (Bruker, 2003 ▶); data reduction: *SAINT*; program(s) used to solve structure: *SHELXS97* (Sheldrick, 2008 ▶); program(s) used to refine structure: *SHELXL97* (Sheldrick, 2008 ▶); molecular graphics: *SHELXTL* (Sheldrick, 2008 ▶); software used to prepare material for publication: *SHELXTL*.

## Supplementary Material

Crystal structure: contains datablocks global, I. DOI: 10.1107/S1600536810049081/pk2286sup1.cif

Structure factors: contains datablocks I. DOI: 10.1107/S1600536810049081/pk2286Isup2.hkl

Additional supplementary materials:  crystallographic information; 3D view; checkCIF report

## Figures and Tables

**Fig. 1.**
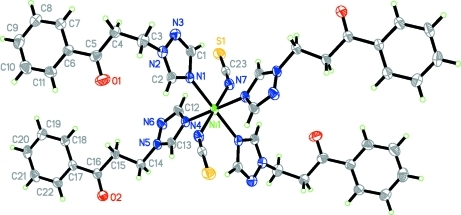
The title complex with atom labeling, shown with 30% probability displacement ellipsoids.

**Fig. 2.**
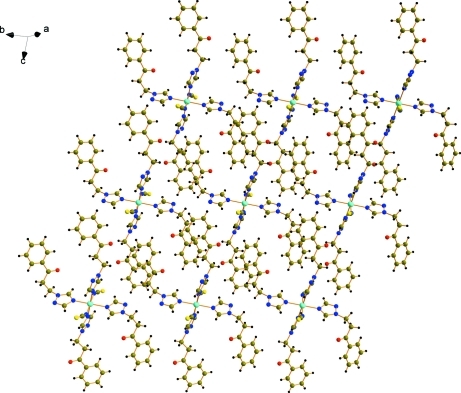
Crystal packing view of compound.

## supplementary crystallographic information

### Comment

Pseudohalide anions N_3_^-^, NCS^-^ and NCO^-^ are known as extremely versatile ligands in coordination chemistry because of their multiple bridging modes (Yue *et al.*, 2008). Recently, we have initiated a research program of synthesizing supramolecules based on pseudohalide and flexible ligands that consist of a propanone unit substituted with an imidazole and a phenyl group (Guo *et al.*, 2007). To further explore this series, we synthesized the title compound, a new Ni^II^ complex based on the mixed ligands thiocyanato and 3-(1*H*-1,2,4-triazol-1-yl)-1-phenylpropan-1-one (*L*), which consists of a propanone unit substituted with an triazole and a phenyl group. The crystal structure of the compound consists of a neutral mononuclear [Ni(*L*)_4_(SCN)_2_] molecule. As shown in Fig. 1, the Ni^II^ centre is coordinated by four N atoms from four *L* ligands, with Ni—N bond lengths in the range 2.103 (1)–2.135 (1) Å, two additional N donor from SCN anion, with a Ni—N bond distance of 2.078 (2) Å. Thus, the coordination polyhedron around the Ni^II^ cation could be best described as a distorted octahedral geometry. Analysis of the crystal packing indicates that there were no hydrogen bond or πi-πi stacking interactions in the crystal structure. (see Fig. 2).

### Experimental

Ni(NO_3_)_2_.6H_2_O (29.1 mg, 0.1 mmol), 3-(1*H*-1,2,4-triazol-1-yl)-1-phenylpropan-1-one (22.3 mg, 0.1 mmol) and NH_4_SCN (7.6 mg, 0.1 mmol) were mixed in a CH_3_CN—H_2_O (20 ml, 1:1 *v*/*v*) solution with vigorous stirring for *ca* 30 min. The resulting solution was filtered and left to stand at room temperature. Green block crystals suitable for X-ray analysis were obtained in 60% yield by slow evaporation of the solvent over a period of 1 week. Analysis, calculated for NiC_46_H_44_N_14_O_4_S_2_: C 56.39, H 4.53, N 20.01; found: C 56.44, H 4.64, N 20.05.

### Refinement

Although all H atoms were visible in difference maps, they were finally placed in geometrically calculated positions, with C—H distances in the range 0.93–0.97 Å, and included in the final refinement in the riding model approximation, with *U*_iso_(H) = 1.2*U*_eq_(C) for aromatic and methylene H atoms.

### Crystal data

**Table:**

[Ni(NCS)_2_(C_11_H_11_N_3_O)_4_]	*Z* = 1
*M**_r_* = 979.78	*F*(000) = 510
Triclinic, *P*1	*D*_x_ = 1.416 Mg m^−^^3^
Hall symbol: -P 1	Mo *K*α radiation, λ = 0.71073 Å
*a* = 7.8067 (10) Å	Cell parameters from 3376 reflections
*b* = 11.8539 (15) Å	θ = 2.7–27.7°
*c* = 13.8179 (17) Å	µ = 0.57 mm^−^^1^
α = 68.907 (2)°	*T* = 293 K
β = 74.765 (2)°	Block, green
γ = 81.687 (2)°	0.32 × 0.28 × 0.22 mm
*V* = 1149.3 (3) Å^3^	

### Data collection

**Table:**

Bruker APEXII CCD area-detector diffractometer	4016 independent reflections
Radiation source: fine-focus sealed tube	3540 reflections with *I* > 2σ(*I*)
graphite	*R*_int_ = 0.015
φ and ω scans	θ_max_ = 25.0°, θ_min_ = 1.8°
Absorption correction: multi-scan (*SADABS*; Sheldrick, 1996)	*h* = −9→8
*T*_min_ = 0.838, *T*_max_ = 0.884	*k* = −14→12
6289 measured reflections	*l* = −16→12

### Refinement

**Table:**

Refinement on *F*^2^	Primary atom site location: structure-invariant direct methods
Least-squares matrix: full	Secondary atom site location: difference Fourier map
*R*[*F*^2^ > 2σ(*F*^2^)] = 0.029	Hydrogen site location: inferred from neighbouring sites
*wR*(*F*^2^) = 0.080	H-atom parameters constrained
*S* = 1.06	*w* = 1/[σ^2^(*F*_o_^2^) + (0.040*P*)^2^ + 0.2997*P*] where *P* = (*F*_o_^2^ + 2*F*_c_^2^)/3
4016 reflections	(Δ/σ)_max_ = 0.001
304 parameters	Δρ_max_ = 0.38 e Å^−^^3^
1 restraint	Δρ_min_ = −0.34 e Å^−^^3^

### Special details

**Table:**

Geometry. All e.s.d.'s (except the e.s.d. in the dihedral angle between two l.s. planes) are estimated using the full covariance matrix. The cell e.s.d.'s are taken into account individually in the estimation of e.s.d.'s in distances, angles and torsion angles; correlations between e.s.d.'s in cell parameters are only used when they are defined by crystal symmetry. An approximate (isotropic) treatment of cell e.s.d.'s is used for estimating e.s.d.'s involving l.s. planes.
Refinement. Refinement of *F*^2^ against ALL reflections. The weighted *R*-factor *wR* and goodness of fit *S* are based on *F*^2^, conventional *R*-factors *R* are based on *F*, with *F* set to zero for negative *F*^2^. The threshold expression of *F*^2^ > 2σ(*F*^2^) is used only for calculating *R*-factors(gt) *etc*. and is not relevant to the choice of reflections for refinement. *R*-factors based on *F*^2^ are statistically about twice as large as those based on *F*, and *R*- factors based on ALL data will be even larger.

### Fractional atomic coordinates and isotropic or equivalent isotropic displacement parameters (Å^2^)

**Table:**

	*x*	*y*	*z*	*U*_iso_*/*U*_eq_	
Ni1	0.0000	0.0000	1.0000	0.03496 (10)	
S1	0.33805 (8)	0.17536 (5)	1.13687 (5)	0.06495 (17)	
O1	−0.2740 (2)	0.43272 (14)	0.66743 (14)	0.0764 (5)	
O2	0.7081 (2)	0.11988 (13)	0.41955 (11)	0.0655 (4)	
N1	−0.13110 (18)	0.17612 (12)	0.96907 (11)	0.0392 (3)	
N2	−0.3141 (2)	0.33084 (13)	0.91887 (12)	0.0452 (4)	
N3	−0.2531 (2)	0.35006 (14)	0.99458 (13)	0.0532 (4)	
N4	0.22222 (18)	0.06368 (13)	0.87523 (10)	0.0396 (3)	
N5	0.42461 (19)	0.09149 (13)	0.72860 (11)	0.0413 (3)	
N6	0.4816 (2)	0.14708 (15)	0.78321 (12)	0.0524 (4)	
N7	0.1164 (2)	0.03338 (14)	1.10545 (12)	0.0463 (4)	
C1	−0.1443 (3)	0.25495 (17)	1.02134 (14)	0.0482 (4)	
H1	−0.0805	0.2426	1.0728	0.058*	
C2	−0.2417 (2)	0.22777 (16)	0.90504 (14)	0.0433 (4)	
H2	−0.2650	0.1963	0.8574	0.052*	
C3	−0.4513 (3)	0.41348 (18)	0.87177 (18)	0.0575 (5)	
H3A	−0.5452	0.4287	0.9282	0.069*	
H3B	−0.5027	0.3749	0.8357	0.069*	
C4	−0.3814 (3)	0.53338 (17)	0.79313 (15)	0.0517 (5)	
H4A	−0.4803	0.5932	0.7850	0.062*	
H4B	−0.3006	0.5603	0.8219	0.062*	
C5	−0.2859 (3)	0.52697 (17)	0.68498 (17)	0.0512 (5)	
C6	−0.2094 (3)	0.63838 (17)	0.59972 (15)	0.0478 (4)	
C7	−0.2128 (3)	0.74666 (18)	0.61733 (17)	0.0589 (5)	
H7	−0.2664	0.7521	0.6842	0.071*	
C8	−0.1372 (3)	0.8465 (2)	0.53641 (19)	0.0696 (6)	
H8	−0.1401	0.9186	0.5493	0.084*	
C9	−0.0584 (3)	0.8405 (2)	0.43801 (19)	0.0694 (6)	
H9	−0.0070	0.9081	0.3840	0.083*	
C10	−0.0551 (3)	0.7347 (2)	0.41887 (19)	0.0725 (7)	
H10	−0.0023	0.7306	0.3514	0.087*	
C11	−0.1297 (3)	0.6340 (2)	0.49890 (17)	0.0618 (6)	
H11	−0.1263	0.5624	0.4851	0.074*	
C12	0.3558 (3)	0.12746 (18)	0.87042 (14)	0.0501 (5)	
H12	0.3584	0.1553	0.9249	0.060*	
C13	0.2719 (2)	0.04232 (16)	0.78431 (13)	0.0430 (4)	
H13	0.2086	−0.0012	0.7625	0.052*	
C14	0.5325 (3)	0.08618 (17)	0.62641 (14)	0.0493 (5)	
H14A	0.4685	0.0474	0.5967	0.059*	
H14B	0.6421	0.0382	0.6369	0.059*	
C15	0.5753 (2)	0.21197 (16)	0.54981 (13)	0.0448 (4)	
H15A	0.4652	0.2606	0.5427	0.054*	
H15B	0.6432	0.2490	0.5789	0.054*	
C16	0.6796 (2)	0.21220 (17)	0.44120 (14)	0.0433 (4)	
C17	0.7446 (2)	0.32998 (16)	0.36077 (13)	0.0409 (4)	
C18	0.6985 (3)	0.43852 (18)	0.37917 (15)	0.0540 (5)	
H18	0.6274	0.4396	0.4445	0.065*	
C19	0.7573 (3)	0.54573 (19)	0.30103 (17)	0.0640 (6)	
H19	0.7237	0.6188	0.3134	0.077*	
C20	0.8645 (3)	0.5447 (2)	0.20578 (16)	0.0605 (6)	
H20	0.9041	0.6170	0.1535	0.073*	
C21	0.9141 (3)	0.4374 (2)	0.18690 (16)	0.0633 (6)	
H21	0.9881	0.4368	0.1222	0.076*	
C22	0.8541 (3)	0.33028 (19)	0.26387 (15)	0.0549 (5)	
H22	0.8873	0.2577	0.2506	0.066*	
C23	0.2074 (2)	0.09092 (16)	1.12141 (13)	0.0401 (4)	

### Atomic displacement parameters (Å^2^)

**Table:**

	*U*^11^	*U*^22^	*U*^33^	*U*^12^	*U*^13^	*U*^23^
Ni1	0.03343 (17)	0.03979 (18)	0.02712 (16)	−0.01089 (12)	−0.00462 (12)	−0.00422 (12)
S1	0.0753 (4)	0.0712 (4)	0.0618 (3)	−0.0221 (3)	−0.0177 (3)	−0.0299 (3)
O1	0.0911 (12)	0.0527 (9)	0.0871 (11)	−0.0029 (8)	−0.0070 (9)	−0.0353 (8)
O2	0.0860 (11)	0.0510 (8)	0.0485 (8)	−0.0119 (7)	0.0079 (7)	−0.0171 (7)
N1	0.0398 (8)	0.0403 (8)	0.0326 (7)	−0.0093 (6)	−0.0067 (6)	−0.0048 (6)
N2	0.0433 (8)	0.0441 (8)	0.0441 (8)	−0.0059 (7)	−0.0123 (7)	−0.0070 (7)
N3	0.0634 (10)	0.0478 (9)	0.0490 (9)	−0.0007 (8)	−0.0173 (8)	−0.0148 (7)
N4	0.0383 (8)	0.0434 (8)	0.0313 (7)	−0.0106 (6)	−0.0035 (6)	−0.0059 (6)
N5	0.0421 (8)	0.0440 (8)	0.0316 (7)	−0.0120 (6)	−0.0004 (6)	−0.0074 (6)
N6	0.0522 (9)	0.0625 (10)	0.0407 (8)	−0.0271 (8)	0.0016 (7)	−0.0149 (7)
N7	0.0434 (8)	0.0555 (9)	0.0386 (8)	−0.0095 (7)	−0.0108 (7)	−0.0106 (7)
C1	0.0595 (12)	0.0468 (10)	0.0389 (10)	−0.0058 (9)	−0.0163 (9)	−0.0103 (8)
C2	0.0427 (10)	0.0456 (10)	0.0390 (9)	−0.0107 (8)	−0.0085 (8)	−0.0089 (8)
C3	0.0442 (11)	0.0561 (12)	0.0645 (13)	0.0005 (9)	−0.0162 (10)	−0.0099 (10)
C4	0.0539 (11)	0.0465 (10)	0.0526 (11)	0.0065 (9)	−0.0187 (9)	−0.0131 (9)
C5	0.0485 (11)	0.0462 (11)	0.0617 (12)	0.0077 (8)	−0.0199 (9)	−0.0204 (9)
C6	0.0481 (11)	0.0468 (10)	0.0494 (11)	0.0110 (8)	−0.0187 (9)	−0.0169 (8)
C7	0.0749 (14)	0.0495 (11)	0.0498 (11)	0.0059 (10)	−0.0145 (10)	−0.0172 (9)
C8	0.0873 (17)	0.0482 (12)	0.0687 (15)	0.0031 (11)	−0.0207 (13)	−0.0149 (11)
C9	0.0619 (14)	0.0622 (14)	0.0656 (15)	0.0035 (11)	−0.0142 (11)	−0.0027 (11)
C10	0.0664 (15)	0.0860 (18)	0.0514 (13)	0.0121 (13)	−0.0045 (11)	−0.0196 (12)
C11	0.0651 (13)	0.0624 (13)	0.0596 (13)	0.0122 (11)	−0.0150 (11)	−0.0282 (11)
C12	0.0558 (11)	0.0570 (11)	0.0366 (9)	−0.0258 (9)	0.0021 (8)	−0.0152 (8)
C13	0.0409 (10)	0.0501 (10)	0.0346 (9)	−0.0159 (8)	−0.0054 (7)	−0.0075 (8)
C14	0.0524 (11)	0.0510 (11)	0.0353 (9)	−0.0089 (9)	0.0050 (8)	−0.0120 (8)
C15	0.0436 (10)	0.0480 (10)	0.0337 (9)	−0.0043 (8)	−0.0008 (7)	−0.0080 (8)
C16	0.0409 (10)	0.0480 (10)	0.0361 (9)	−0.0038 (8)	−0.0054 (7)	−0.0106 (8)
C17	0.0383 (9)	0.0477 (10)	0.0323 (9)	−0.0046 (8)	−0.0070 (7)	−0.0082 (7)
C18	0.0627 (12)	0.0512 (11)	0.0382 (10)	−0.0030 (9)	−0.0022 (9)	−0.0098 (8)
C19	0.0799 (16)	0.0478 (11)	0.0563 (13)	−0.0061 (11)	−0.0133 (11)	−0.0088 (10)
C20	0.0604 (13)	0.0596 (13)	0.0482 (12)	−0.0217 (10)	−0.0102 (10)	0.0027 (10)
C21	0.0624 (13)	0.0771 (15)	0.0361 (10)	−0.0192 (11)	0.0063 (9)	−0.0088 (10)
C22	0.0611 (12)	0.0584 (12)	0.0385 (10)	−0.0109 (10)	0.0022 (9)	−0.0152 (9)
C23	0.0417 (9)	0.0467 (10)	0.0299 (8)	−0.0030 (7)	−0.0084 (7)	−0.0101 (7)

### Geometric parameters (Å, °)

**Table:**

Ni1—N7^i^	2.0783 (15)	C6—C7	1.385 (3)
Ni1—N7	2.0783 (15)	C6—C11	1.385 (3)
Ni1—N4	2.1028 (13)	C7—C8	1.379 (3)
Ni1—N4^i^	2.1028 (13)	C7—H7	0.9300
Ni1—N1	2.1351 (14)	C8—C9	1.360 (3)
Ni1—N1^i^	2.1351 (14)	C8—H8	0.9300
S1—C23	1.6282 (19)	C9—C10	1.368 (4)
O1—C5	1.212 (2)	C9—H9	0.9300
O2—C16	1.211 (2)	C10—C11	1.378 (3)
N1—C2	1.327 (2)	C10—H10	0.9300
N1—C1	1.351 (2)	C11—H11	0.9300
N2—C2	1.327 (2)	C12—H12	0.9300
N2—N3	1.355 (2)	C13—H13	0.9300
N2—C3	1.461 (2)	C14—C15	1.509 (2)
N3—C1	1.311 (2)	C14—H14A	0.9700
N4—C13	1.318 (2)	C14—H14B	0.9700
N4—C12	1.349 (2)	C15—C16	1.507 (2)
N5—C13	1.322 (2)	C15—H15A	0.9700
N5—N6	1.349 (2)	C15—H15B	0.9700
N5—C14	1.460 (2)	C16—C17	1.495 (2)
N6—C12	1.309 (2)	C17—C18	1.379 (3)
N7—C23	1.162 (2)	C17—C22	1.385 (2)
C1—H1	0.9300	C18—C19	1.383 (3)
C2—H2	0.9300	C18—H18	0.9300
C3—C4	1.516 (3)	C19—C20	1.365 (3)
C3—H3A	0.9700	C19—H19	0.9300
C3—H3B	0.9700	C20—C21	1.371 (3)
C4—C5	1.508 (3)	C20—H20	0.9300
C4—H4A	0.9700	C21—C22	1.379 (3)
C4—H4B	0.9700	C21—H21	0.9300
C5—C6	1.492 (3)	C22—H22	0.9300
			
N7^i^—Ni1—N7	180.0	C8—C7—H7	119.8
N7^i^—Ni1—N4	89.53 (6)	C6—C7—H7	119.8
N7—Ni1—N4	90.47 (6)	C9—C8—C7	120.7 (2)
N7^i^—Ni1—N4^i^	90.47 (6)	C9—C8—H8	119.7
N7—Ni1—N4^i^	89.53 (6)	C7—C8—H8	119.7
N4—Ni1—N4^i^	180.0	C8—C9—C10	119.7 (2)
N7^i^—Ni1—N1	90.38 (6)	C8—C9—H9	120.2
N7—Ni1—N1	89.62 (6)	C10—C9—H9	120.2
N4—Ni1—N1	92.47 (5)	C9—C10—C11	120.4 (2)
N4^i^—Ni1—N1	87.53 (5)	C9—C10—H10	119.8
N7^i^—Ni1—N1^i^	89.62 (6)	C11—C10—H10	119.8
N7—Ni1—N1^i^	90.38 (6)	C10—C11—C6	120.6 (2)
N4—Ni1—N1^i^	87.53 (5)	C10—C11—H11	119.7
N4^i^—Ni1—N1^i^	92.47 (5)	C6—C11—H11	119.7
N1—Ni1—N1^i^	180.0	N6—C12—N4	114.85 (17)
C2—N1—C1	102.31 (15)	N6—C12—H12	122.6
C2—N1—Ni1	128.52 (13)	N4—C12—H12	122.6
C1—N1—Ni1	128.48 (12)	N4—C13—N5	110.34 (16)
C2—N2—N3	110.13 (15)	N4—C13—H13	124.8
C2—N2—C3	129.49 (17)	N5—C13—H13	124.8
N3—N2—C3	120.18 (16)	N5—C14—C15	110.42 (15)
C1—N3—N2	102.04 (15)	N5—C14—H14A	109.6
C13—N4—C12	102.53 (14)	C15—C14—H14A	109.6
C13—N4—Ni1	127.68 (12)	N5—C14—H14B	109.6
C12—N4—Ni1	129.61 (12)	C15—C14—H14B	109.6
C13—N5—N6	109.87 (14)	H14A—C14—H14B	108.1
C13—N5—C14	129.32 (16)	C16—C15—C14	112.64 (15)
N6—N5—C14	120.71 (14)	C16—C15—H15A	109.1
C12—N6—N5	102.40 (14)	C14—C15—H15A	109.1
C23—N7—Ni1	149.53 (14)	C16—C15—H15B	109.1
N3—C1—N1	115.41 (17)	C14—C15—H15B	109.1
N3—C1—H1	122.3	H15A—C15—H15B	107.8
N1—C1—H1	122.3	O2—C16—C17	121.11 (16)
N2—C2—N1	110.11 (17)	O2—C16—C15	120.90 (16)
N2—C2—H2	124.9	C17—C16—C15	117.98 (16)
N1—C2—H2	124.9	C18—C17—C22	118.80 (17)
N2—C3—C4	113.09 (16)	C18—C17—C16	122.40 (16)
N2—C3—H3A	109.0	C22—C17—C16	118.79 (17)
C4—C3—H3A	109.0	C17—C18—C19	120.34 (19)
N2—C3—H3B	109.0	C17—C18—H18	119.8
C4—C3—H3B	109.0	C19—C18—H18	119.8
H3A—C3—H3B	107.8	C20—C19—C18	120.2 (2)
C5—C4—C3	113.39 (17)	C20—C19—H19	119.9
C5—C4—H4A	108.9	C18—C19—H19	119.9
C3—C4—H4A	108.9	C19—C20—C21	120.16 (19)
C5—C4—H4B	108.9	C19—C20—H20	119.9
C3—C4—H4B	108.9	C21—C20—H20	119.9
H4A—C4—H4B	107.7	C20—C21—C22	119.97 (19)
O1—C5—C6	120.63 (19)	C20—C21—H21	120.0
O1—C5—C4	120.17 (19)	C22—C21—H21	120.0
C6—C5—C4	119.19 (17)	C21—C22—C17	120.5 (2)
C7—C6—C11	118.2 (2)	C21—C22—H22	119.8
C7—C6—C5	122.69 (18)	C17—C22—H22	119.8
C11—C6—C5	119.16 (18)	N7—C23—S1	176.90 (16)
C8—C7—C6	120.5 (2)		
			
N7^i^—Ni1—N1—C2	0.68 (14)	O1—C5—C6—C7	−177.0 (2)
N7—Ni1—N1—C2	−179.32 (14)	C4—C5—C6—C7	4.0 (3)
N4—Ni1—N1—C2	90.22 (14)	O1—C5—C6—C11	2.2 (3)
N4^i^—Ni1—N1—C2	−89.78 (14)	C4—C5—C6—C11	−176.77 (18)
N7^i^—Ni1—N1—C1	169.37 (15)	C11—C6—C7—C8	−0.5 (3)
N7—Ni1—N1—C1	−10.63 (15)	C5—C6—C7—C8	178.7 (2)
N4—Ni1—N1—C1	−101.08 (15)	C6—C7—C8—C9	0.1 (4)
N4^i^—Ni1—N1—C1	78.92 (15)	C7—C8—C9—C10	0.4 (4)
C2—N2—N3—C1	0.4 (2)	C8—C9—C10—C11	−0.6 (4)
C3—N2—N3—C1	175.77 (16)	C9—C10—C11—C6	0.2 (4)
N7^i^—Ni1—N4—C13	−19.91 (15)	C7—C6—C11—C10	0.3 (3)
N7—Ni1—N4—C13	160.09 (15)	C5—C6—C11—C10	−178.9 (2)
N1—Ni1—N4—C13	−110.27 (15)	N5—N6—C12—N4	−0.2 (2)
N1^i^—Ni1—N4—C13	69.73 (15)	C13—N4—C12—N6	0.5 (2)
N7^i^—Ni1—N4—C12	165.83 (16)	Ni1—N4—C12—N6	175.88 (13)
N7—Ni1—N4—C12	−14.17 (16)	C12—N4—C13—N5	−0.6 (2)
N1—Ni1—N4—C12	75.48 (16)	Ni1—N4—C13—N5	−176.09 (11)
N1^i^—Ni1—N4—C12	−104.52 (16)	N6—N5—C13—N4	0.5 (2)
C13—N5—N6—C12	−0.2 (2)	C14—N5—C13—N4	176.90 (16)
C14—N5—N6—C12	−176.91 (17)	C13—N5—C14—C15	128.03 (19)
N4—Ni1—N7—C23	32.2 (3)	N6—N5—C14—C15	−55.9 (2)
N4^i^—Ni1—N7—C23	−147.8 (3)	N5—C14—C15—C16	−177.37 (15)
N1—Ni1—N7—C23	−60.3 (3)	C14—C15—C16—O2	6.2 (3)
N1^i^—Ni1—N7—C23	119.7 (3)	C14—C15—C16—C17	−174.59 (16)
N2—N3—C1—N1	−0.2 (2)	O2—C16—C17—C18	173.0 (2)
C2—N1—C1—N3	−0.1 (2)	C15—C16—C17—C18	−6.3 (3)
Ni1—N1—C1—N3	−171.03 (12)	O2—C16—C17—C22	−6.5 (3)
N3—N2—C2—N1	−0.5 (2)	C15—C16—C17—C22	174.30 (17)
C3—N2—C2—N1	−175.28 (16)	C22—C17—C18—C19	1.5 (3)
C1—N1—C2—N2	0.33 (19)	C16—C17—C18—C19	−177.93 (19)
Ni1—N1—C2—N2	171.29 (11)	C17—C18—C19—C20	−1.3 (3)
C2—N2—C3—C4	−110.8 (2)	C18—C19—C20—C21	0.2 (4)
N3—N2—C3—C4	74.8 (2)	C19—C20—C21—C22	0.6 (4)
N2—C3—C4—C5	79.4 (2)	C20—C21—C22—C17	−0.4 (3)
C3—C4—C5—O1	1.3 (3)	C18—C17—C22—C21	−0.7 (3)
C3—C4—C5—C6	−179.74 (16)	C16—C17—C22—C21	178.80 (19)

Symmetry codes: (i) −*x*, −*y*, −*z*+2.
